# Draft Genome Sequences of Colistin-Resistant and *mcr-1.1-*Carrying Escherichia coli Strains Isolated from Irrigation Water

**DOI:** 10.1128/MRA.00120-21

**Published:** 2021-04-08

**Authors:** Nivin A. Nasser, David Mann, Shaoting Li, Xiangyu Deng, Issmat I. Kassem

**Affiliations:** a Center for Food Safety, Department of Food Science and Technology, University of Georgia, Griffin, Georgia, USA; b Department of Nutrition and Food Sciences, Faculty of Agricultural and Food Sciences, American University of Beirut (AUB), Beirut, Lebanon; University of Maryland School of Medicine

## Abstract

Colistin has been classified as a highest priority critically important antibiotic. However, the emergence of mobile colistin resistance (*mcr*) genes has threatened the therapeutic uses of colistin. Here, we report the draft genome sequences, antibiotic resistance genes, and sequence types (STs) of two multidrug-resistant and *mcr-1.1*-positive Escherichia coli strains isolated from irrigation water.

## Abstract (precis)

Colistin has been classified as a highest priority clinically important antibiotic. However, the emergence of mobile colistin resistance (*mcr*) genes has threatened the therapeutic uses of colistin. Here, we report the draft genome sequences, antibiotic resistance genes, and sequence types (STs) of two multidrug-resistant and *mcr-1.1*-positive Escherichia coli strains isolated from irrigation water.

## ANNOUNCEMENT

The global dissemination of mobile colistin resistance (*mcr*) genes has threatened the efficacy of colistin (1). Previously, we reported the occurrence of colistin-resistant and *mcr-1-*positive Escherichia coli strains in irrigation water in Lebanon (1–3). The bacteria were isolated from composite water samples (1 liter) collected from a major agricultural area. The samples were filtered using 0.22-μm Millipore membranes that were placed onto selective RAPID'E. coli 2 agar (Bio-Rad, USA) supplemented with 4 μg/ml colistin (Sigma-Aldrich, USA) (1, 4). The plates were incubated at 37°C under aerobic conditions for 18 to 24 h, and colonies that exhibited an E. coli phenotype were purified and further identified using PCR analysis (1, 5). Two E. coli strains that exhibited relatively high colistin resistance (Table 1) were selected for whole-genome sequencing (WGS) analysis.

**TABLE 1:** Genome properties and antibiotic resistance profile of the E. coli isolates harboring *mcr-1.1*, isolated from irrigation water in the Beqaa Valley, a major agricultural area in Lebanon

E. coli isolate	SRA accession no. of raw sequences	GenBank accession no. of assembled genomes	Genome size (bp)	Total no. of reads	No. of contigs	*N*_50_ (bp)	*L*_50_	Genome coverage (×)	GC content (%)	Colistin MIC (μg/ml)^*a*^	Resistance to selected antibiotics^*b*^:	Total no. of antibiotic resistance genes detected by WGS^*c*^	Acquired antibiotic resistance genes detected by WGS	Sequence type
C3	SRX7741063	JADBJE000000000	5,474,603	204,635,755	182	161,363	182	41	50.7	64	R: PEN, AMP, FEP, CTX, LEX, CFM, DOR, GEN, KAN, STR, TET, CIP, NOR, SXT, CHL IR: MEN S: AMC	15	*acc(3)-IId*; *aadA5*; *ant(3″)-Ia*; *aph(*3*″)-Ib*; *aph(*3*′)-Ia*; *aph(*6*)-Id*; *bla*_TEM-1B_; *dfrA17*; *erm42*; *floR*; ***mcr-1.1***; *mdfA*; *mphA*; *sul2*; *tetA*	162
C5	SRX7741065	JADBJD000000000	5,014,062	445,089,924	304	97,303	18	89	50.8	64	R: PEN, AMP, AMC, FEP, CTX, LEX, CFM, KAN, STR, TET, CIP, NOR, SXT IR: CHL S: DOR, MEM, GEN	17	*aadA2*; *ant(*3*″)-Ia*; *aph*(*3*″)*-Ib*; *aph(*3*′)-Ia*; *aph6-Id*; *bla*_CTX-M-3_; *bla*_TEM-1B_; *dfrA14*; *dfrA1_10*; *floR*; *fosA3*; ***mcr-1.1***; *mdfA*; *mphA*; *sul1*; *sul3*; *tetA*	10

a The colistin MIC was quantified using the broth microdilution method. Resistance to other antibiotics was determined using the disk diffusion assay and the Clinical and Laboratory Standards Institute (CLSI) guidelines (12).

b R, resistance; IR, intermediate resistance; S, susceptibility; PEN, penicillin; AMP, ampicillin; AMC, amoxicillin plus clavulanic acid; FEP, cefepime; CTX, cefotaxime; LEX, cephalexin; CFM, cefixime; DOR, doripenem; MEM, meropenem; GEN, gentamicin; KAN, kanamycin; STR, streptomycin; TET, tetracycline; CIP, ciprofloxacin; NOR, norfloxacin; SXT, trimethoprim-sulfamethoxazole; CHL, chloramphenicol. The antibiotics in the resistance profiles are arranged according to the order of antibiotics/classes listed in the CLSI guidelines (12).

c WGS, whole-genome sequencing.

The E. coli strains were cultured on RAPID'E. coli 2 agar as described above. Using inoculation loops, colonies were transferred from the agar plates for genomic DNA isolation and quantification using the QiaAmp DNA minikit (Qiagen, USA) and the Qubit double-stranded DNA (dsDNA) broad-range (BR) assay kit (Invitrogen, USA), respectively, as described in the manufacturers’ protocols. The Nextera XT DNA library preparation kit and the Qubit dsDNA high-sensitivity (HS) broad-range (BR) assay kit (Invitrogen) were used to prepare and determine the concentrations of the sample libraries, respectively (6). The libraries were diluted and denatured according to protocol A in the Illumina “Denature and Dilute Libraries Guide” (https://support.illumina.com/content/dam/illumina-support/documents/documentation/system_documentation/miseq/miseq-denature-dilute-libraries-guide-15039740-10.pdf) and loaded into the MiSeq reagent cartridge (MiSeq reagent kit v2, 300 cycles) (6). Sequencing was then performed with a MiSeq sequencer (Illumina, USA) using the paired-end sequencing strategy (2 × 150 bp). Low-quality reads were removed with Trimmomatic v0.36 (7). The leading three and the trailing three nucleotides were removed from the reads, and a 4-nucleotide sliding window was used to also remove nucleotides from the 3′ ends when the average Phred score dropped below 20. Reads shorter than 75 bp were discarded, and the total number of reads was obtained for each isolate. Draft genome sequences were assembled from the trimmed and filtered reads using the “–careful” option in SPAdes v3.9.0 (8). Contigs shorter than 200 bp were discarded, and the quality of the draft genome was evaluated with QUAST v4.5 (9). Sequence types (STs) were determined using the PubMLST database (https://pubmlst.org/) with the MLST software v2.16.2 (https://github.com/tseemann/mlst) (10). Default parameters were used for all software unless otherwise specified.

The properties of the draft genome sequences are listed in Table 1. Analysis using the ResFinder v3.0 database (11) showed that the E. coli strains carried up to 17 antibiotic resistance genes (Table 1), including *mcr-1.1* and others that encoded resistance to important classes of antibiotics, such as aminoglycosides, diaminopyrimidines, β-lactams, fosfomycin, tetracyclines, fluoroquinolones, and sulfonamides (Table 1). Multilocus sequence type (MLST) analysis revealed that the strains belonged to ST10 and ST162 (Table 1), which have been associated with *mcr-1-*positive E. coli strains isolated from a variety of hosts and niches (13–17).

The draft genome sequences are important for understanding the dissemination of colistin-resistant E. coli strains and plasmid-borne *mcr-1.1* in agricultural waters, which might select for antibiotic resistance determinants that persist in the food chain.

### Data availability.

The raw sequences for the analyzed strains can be found under accession numbers SRX7741063 and SRX7741065, while the assembled genome sequences were deposited under accession numbers JADBJE000000000 (GCA_014892385.1) and JADBJD000000000 (GCA_014892435.1), respectively.
